# Elucidating Spatially-Resolved Changes in Host Signaling During *Plasmodium* Liver-Stage Infection

**DOI:** 10.3389/fcimb.2021.804186

**Published:** 2022-01-17

**Authors:** Elizabeth K. K. Glennon, Tinotenda Tongogara, Veronica I. Primavera, Sophia M. Reeder, Ling Wei, Alexis Kaushansky

**Affiliations:** ^1^ Seattle Children’s Research Institute, Center for Global Infectious Disease Research, Seattle, WA, United States; ^2^ Grinnell College, Grinnell, IA, United States; ^3^ Department of Global Health, University of Washington, Seattle, WA, United States; ^4^ Department of Pediatrics, University of Washington, Seattle, WA, United States; ^5^ Brotman Baty Institute for Precision Medicine, Seattle, WA, United States

**Keywords:** plasmodium, liver stage, mouse model, spatial profiling, kupffer cell

## Abstract

Upon transmission to the human host, *Plasmodium* sporozoites exit the skin, are taken up by the blood stream, and then travel to the liver where they infect and significantly modify a single hepatocyte. Low infection rates within the liver have made proteomic studies of infected hepatocytes challenging, particularly *in vivo*, and existing studies have been largely unable to consider how protein and phosphoprotein differences are altered at different spatial locations within the heterogeneous liver. Using digital spatial profiling, we characterized changes in host signaling during *Plasmodium yoelii* infection *in vivo* without disrupting the liver tissue. Moreover, we measured alterations in protein expression around infected hepatocytes and identified a subset of CD163^+^ Kupffer cells that migrate towards infected cells during infection. These data offer the first insight into the heterogeneous microenvironment that surrounds the infected hepatocyte and provide insights into how the parasite may alter its milieu to influence its survival and modulate immunity.

## Introduction

Upon introduction to the human host by the bite of an infectious mosquito, *Plasmodium* parasites migrate to the liver where they invade a hepatocyte and proceed to develop and replicate. Once parasites complete their development within the liver, thousands of individual merozoites egress from the host hepatocyte and migrate to the bloodstream where they invade erythrocytes and initiate the symptomatic blood stage of infection. The liver is often discussed as a uniform organ, however, factors such as oxygen and nutrient gradients lead to diverse cellular phenotypes and the formation of niches within the tissue (Kietzmann, 2017; Gola et al., 2021). *Plasmodium* parasites traverse multiple hepatocytes before invading one (Mota et al., 2001; Coppi et al., 2007; Loubens et al., 2021) and preferentially invade both particular liver zones (Yang et al., 2021) and hepatocytes with specific phenotypes, such as high ploidy and particular surface receptor compositions (Austin et al., 2014; Kaushansky et al., 2015a). In addition to selecting particular hepatocytes for invasion, parasites modify the host cell throughout their development within the liver, including cell size (Balasubramanian et al., 2019), microtubule and organelle organization (Vijayan et al., 2020), and signaling cascades (Kaushansky et al., 2013a; Glennon et al., 2019).

The liver stage (LS) is a substantial bottleneck in *Plasmodium* infection, making it an attractive point for intervention. Attrition in parasite numbers occurs between injection at the skin, invasion of hepatocytes, and completion of development within the liver (Amino et al., 2006). Heterogeneity among hepatocytes within and between individuals can exacerbate this attrition; susceptibility between even closely related mouse strains varies dramatically (Kaushansky et al., 2015b), and the ability of hepatocytes to support *Plasmodium falciparum* and *Plasmodium vivax* infection varied extensively between individual human donors (Roth et al., 2018). Experiments with genetically attenuated parasites demonstrated that parasites that successfully invade but die before completing LS infection can induce immunity and reduce susceptibility to subsequent infection (Vaughan and Kappe, 2017).

Several global studies have been conducted to understand alterations that occur during LS infection which may be important for the maintenance of infection. Transcriptomic studies have demonstrated extensive changes in host gene expression that vary over the course of infection, however concordance among these studies has been low, perhaps due to differences in hepatocyte origin and time needed to sort infected cells (Albuquerque et al., 2009; LaMonte et al., 2019). We used reverse phase protein array (RPPA) to evaluate changes in host protein and post-translational modifications in an *in vitro* model of *Plasmodium yoelii* infection (Kaushansky et al., 2013a), and to identify proteins that are expressed proteins that are expressed at different levels between hepatocyte populations of differential susceptibility to LS infection (Dembele et al., 2019; Glennon et al., 2019). Several proteins and processes that were identified as altered in infected cells were also found to be important for LS infection [reviewed in (Glennon et al., 2018)]. A small RPPA screen of infected hepatocytes revealed a suppression of p53 levels which was then found to be critical for maintenance of LS infection *in vitro* and *in vivo* (Kaushansky et al., 2013a; Kain et al., 2020). RNA-sequencing of infected hepatocytes revealed an increase in expression of aquaporin-3 (AQP3) and follow-up studies identified AQP3 as essential for infection and implicated it in nutrient acquisition (Posfai et al., 2018). Multiple approaches have been used to elucidate functional regulators of LS infection *in vitro*, including screens based on siRNA, CRISPR/Cas9, and kinase regression (Prudencio et al., 2008; Arang et al., 2017; Raphemot et al., 2019). However, large-scale proteomic studies of liver-stage infection, particularly *in vivo*, have been hindered by low infection rates, on the order of 1% *in vitro* and 0.01% *in vivo* (Prudencio et al., 2011). Additionally, *in vivo* studies traditionally involve sorting infected from uninfected cells and pooling all uninfected cells together, thereby losing the ability to link parasite biology to its microenvironment, or heterogeneity among uninfected cells to their spatial distribution within the liver and position relative to the infected cell.

## Results

**Figure 1:**
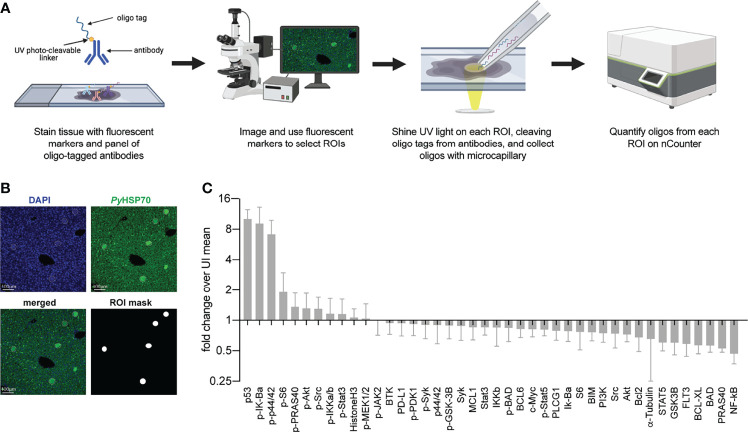
Digital Spatial Profiling facilitates evaluation of proteins and post-translational modifications in *Plasmodium yoelii* infected tissue. **(A)** Schematic of DSP methodology. **(B)** Representative images of fluorescent staining of liver sections from uninfected or *P. yoelii*-infected mice at 44hpi. Parasites are stained with *Py*HSP70 in green and DAPI is shown in blue. Data were pooled from regions of interest indicated by white circles. **(C)** Signal from *P. yoelii*-infected mice normalized to ROI area and to the average signal from uninfected mice. Error bars indicate standard deviation. n = 7-8 mice per group with one pooled ROI measured from each mouse.

To interrogate differences in host cell signaling in intact *Plasmodium*-infected liver tissue we used Digital Spatial Profiling (DSP). DSP interrogates levels of total and phosphorylated proteins in user-defined regions of fixed tissue (Beechem, 2020), thereby preserving spatial information and limiting sample processing that could induce artificial changes. To date, DSP has primarily been used to study heterogeneity within the tumor microenvironment, which has been strongly linked to disease progression and treatment outcomes (Stewart et al., 2020; Wang et al., 2021). Briefly, liver sections are scanned and regions of interest (ROIs) are selected based on staining with fluorescent markers. Slides are incubated with one of several panels of antibodies bound with a photocleavable linker to unique oligonucleotide barcode tags. UV light is shone on the defined ROIs, cleaving the oligo tags from bound antibodies which are collected and quantified using the nCounter system (**Figure 1A**). We infected BALB/c mice with 1 million *P. yoelii* sporozoites and allowed the infection to proceed for 44 hours. Liver sections (4 µm thick) from 7 infected and 8 uninfected mice were stained using an antibody directed against parasite protein *Py*HSP70 that had been covalently linked to Alexa Fluor 488. In parallel with fluorescent staining, liver sections were also incubated with a panel of 42 oligo-tagged antibodies against a variety of host proteins and/or post-translational modifications (**Table S1A**). Slides were imaged and pooled ROIs that encompassed five infected cells (~50 µm diameters) or corresponding uninfected regions were identified (**Figures 1A, B**). Oligos were cleaved and collected from ROIs using the GeoMx Digital Spatial Profiler for quantification. We observed host proteins/post-translational modifications that were both up- and down-regulated in infected regions compared to uninfected mice (**Figure 1C**), some of which have been previously identified as altered upon, or important for, infection (**Table S1A**) (Kaushansky et al., 2013a; Kaushansky et al., 2013b; Douglass et al., 2015; Boonhok et al., 2016; Arang et al., 2017; Glennon et al., 2019; Sharma et al., 2021). Because we conducted multiple DSP runs with tissues on multiple slides, we wanted to compare the reproducibility of our results and identify the contribution of run-to-run variation to the observed variability. Liver sections from infected and uninfected mice were evenly distributed across slides and runs. Comparisons of the fold change in protein levels (infected over uninfected) between two runs (**Figure S1A**), as well as between two slides within a single run (**Figure S1B**), all gave strong linear correlations, suggesting the data from multiple experiments are comparable. Interestingly, the slope of the line between two separate runs (**Figure S1A**) was greater than one, suggesting that comparisons of the magnitude of change between runs should be interpreted with caution.

**Figure 2:**
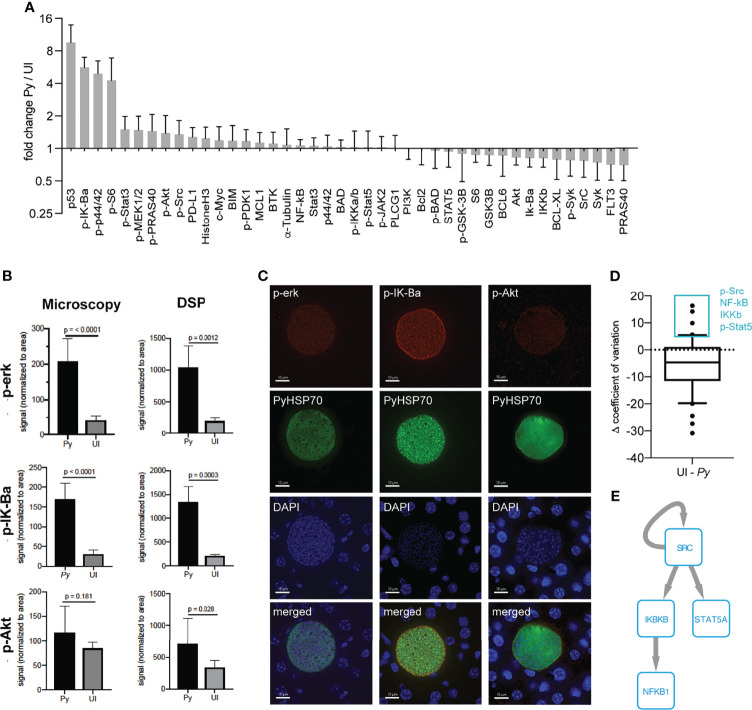
Single infected hepatocytes produce sufficient signal for detection by DSP. **(A)** Signal from 9 infected cells from a single *P. yoelii*-infected mouse normalized to region of interest (ROI) area and to the average signal from 6 uninfected cell regions from a single uninfected mouse. Error bars indicate standard deviation. **(B)** ROI-area-normalized signal from single infected and uninfected ROIs as measured by DSP (from panel **A**) and single antibody staining fluorescent microscopy (n = 6 ROIs each from a single infected or uninfected mouse). **(C)** Representative images of infected cells were taken at a total magnification of 400x at 44hpi. **(D)** Difference in coefficient of variation between uninfected and single infected ROIs (from panel **A**) for each antibody. Box plot encompasses 10^th^-90^th^ percentile. Antibodies within the 90^th^ percentile which showed less variation in infected (*Py*) than in uninfected ROIs are boxed in blue. **(E)** Phosphosignaling network constructed from proteins with lower variation in infected ROIs. Arrows indicate direct phosphorylation events.

We next asked if we could reliably detect changes in host protein levels in single infected cells by DSP. We hypothesized that the enlarged size of infected hepatocytes at 44hpi (approximately 50µm in diameter) might allow us to reliably detect changes in host proteins at the single cell level. The same panel of antibodies (**Table S1A**) was used to detect host protein levels in single infected-cell ROIs from a single infected mouse and in identical sized ROIs encompassing roughly 10 uninfected cells from a single uninfected mouse. Plotting the average of multiple infected single ROIs against the infected pooled ROI, for each antibody, gave a strong linear correlation (**Figure S1C**). The same was found for the comparable uninfected ROIs (**Figure S1D**), suggesting we have sufficient resolution to detect changes in host protein levels within single infected cells for these enlarged, infected cells. Upon examining the fold change between infected and uninfected single ROIs we observed a high degree of similarity between our pooled and single ROIs. The same top four proteins were seen in both pooled and single ROIs (**Figure 1A** and **Figure 2A**), suggesting that the increase detected in the pooled ROIs was not due to a small population of high-expressing cells but may in fact be a feature of multiple infected hepatocytes. As an orthologous approach, we used immunofluorescent microscopy to evaluate several proteins that exhibited differential levels in infected and uninfected cells. Patterns of changes in protein levels between infected and uninfected ROIs were comparable as measured by DSP and by single fluorescent antibody staining (**Figures 2B, C** and **Figure S1E**). We observed localization of multiple host proteins within the parasitophorous vacuole. One possible explanation is nonspecific binding to parasite proteins, which cannot be ruled out using this approach. Another is uptake of host cell cytosol by the parasite, as has been described in blood stage *Plasmodium* parasites and in multiple stages of the related apicomplexan parasite *Toxoplasma gondii* (Hanssen et al., 2012; Dou et al., 2014; Jonscher et al., 2019; Kannan et al., 2021). Distinguishing between these two possibilities, and further exploring the potential for cytosol uptake by the liver stage parasite, is an interesting area for future study.

We used the ability to make measurements in single infected cells to ask how host protein levels varied in single infected cells when compared to uninfected ROIs (**Figure 2D** and **Table S2**). We reasoned that proteins that exhibited substantially less variation between infected cells might represent features that are selected or tuned by the parasite to facilitate its survival and/or development. When examining the difference in variation between infected and uninfected ROIs for our panel of antibodies, we observed a trend towards increased variation in infected ROIs (**Figure 2D**). This is likely explained by the masking of single cell variation within the uninfected ROI, which encompasses roughly ten cells. Despite the skewedness of the data, several total and phosphorylated proteins (p-Src, NF-kB, IKKb, and p-Stat5) exhibited more variation in the uninfected ROI than in single infected cells (> 90^th^ percentile) (**Figure 2D**). While data from more individual cells is required for a robust assessment of the distribution of given host signals, these data can be used for hypothesis generation. To investigate whether proteins with less variation in infected cells might act as part of a connected network, we reconstructed a phosphosignaling network using a database of known kinase target sequences (**Figure 2E**). Network reconstruction revealed that proteins with lower variation in infected cells can directly interact with each other *via* phosphorylation, suggesting they might regulate a common process important for infection and could be a target of parasite selection and/or manipulation. Of particular interest, the two kinases, Src and IKKb, were predicted to regulate LS infection; we have previously shown that shRNA-mediated knockdown of IKBKB reduces parasite numbers *in vitro* (Arang et al., 2017). Increased variation in infected cells could be due to host cell and/or parasite-intrinsic heterogeneity, or due to the influence of different local microenvironments within the liver. These network-level hypotheses represent interesting areas for future investigation.

**Figure 3:**
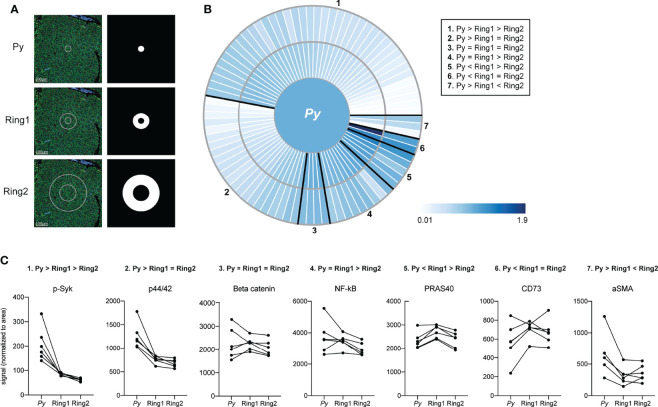
Signals are altered in rings surrounding *Plasmodium*-infected hepatocytes. **(A)** Representative images of fluorescent staining of liver sections and region of interest (ROI) masks. **(B)** Heat maps showing fold change in protein levels between ring ROIs. Signal was normalized to ROI area and to the infected cell ROI, set at 1. Proteins showing similar relative patterns of expression across rings are grouped together and outlined in black. **(C)** Area normalized signal of antibodies illustrative of each spatial pattern. Lines connect matched infected cell and ring ROIs. n= 6 matched ROI sets from a single mouse.

In addition to investigating signaling within the infected cells, we also asked how and if the environment that surrounds the parasite differed from more parasite-distal areas within the liver. Using concentric ring ROIs matched with each LS parasite, we measured protein levels in infected cells, proximal uninfected cells (Ring1), and distal uninfected cells (Ring2) surrounding each parasite (**Figure 3A**). In addition to the original antibody panel, we included a second panel encompassing proteins expressed on various immune cells (**Table S1B**). Protein levels in Ring1 and Ring2 were compared to those in their paired infected ROI and fell into clusters based on spatial patterns of relative expression (**Figures 3B, C** and **Table S3**). We were particularly interested in proteins with higher levels in Ring1 compared to Ring2 (**Table S4**) and theorized that these might be indicative of either (1) immune cell infiltration towards the infected hepatocyte, (2) selection of a cellular niche on a fine scale, or (3) neighboring cells responding to signals emanating out from the infected cell. Of the proteins with significantly higher levels in Ring1 compared to Ring2, several immune cell surface markers, all of which have been described on macrophages (PD-L1, B7-H3, CD68, CD163) (Krenkel and Tacke, 2017; Mao et al., 2017; Sun et al., 2018), were the most heavily upregulated (**Table S4**), leading us to investigate the distribution of macrophages around the parasite.

**Figure 4:**
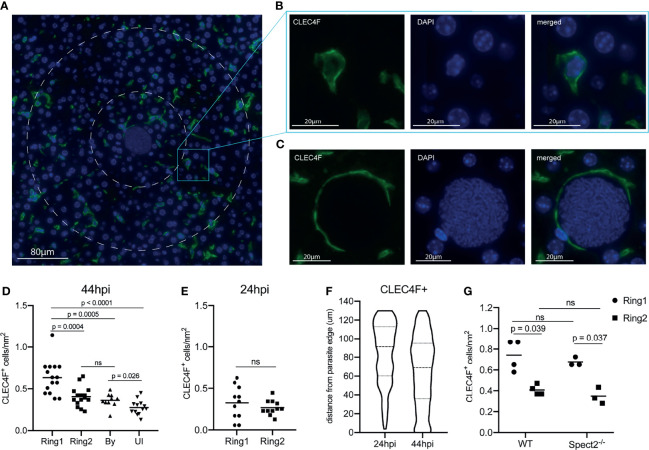
A high density of macrophages surrounds *Plasmodium* infected hepatocytes. **(A)** Representative image of CLEC4F^+^ staining within an infected liver. Nuclear DAPI staining is shown in blue, CLEC4F in green. Ring1 and Ring2 outer boundaries are indicated by dashed white circles. **(B)** CLEC4F staining of a single cell within Ring2 **(C)** Representative image of a CLEC4F^+^ cell in close proximity to a parasite at 44hpi. **(D)** Number of CLEC4F^+^ cells normalized by area in Ring1, Ring2, bystander (By) tissue, and uninfected (UI) tissue at 44hpi. 3-6 matched ROI sets were counted from each of three mice. **(E)** Number of CLEC4F^+^ cells normalized by area in Ring1 and Ring2 at 24hpi. **(F)** Violin plot showing distribution of CLEC4F^+^ cells binned by distance from parasite edge at 24 and 44hpi. **(G)** Levels of CLEC4F^+^ cells in Ring1 and Ring2 around wild type (WT) and Spect2^-/-^ parasites at 44hpi. ns, not significant.

Macrophages populations within the liver can be resident or monocyte-derived. Kupffer cells, the resident liver macrophage, are the most prevalent non-parenchymal cell in the liver, making up about 35% of total cells (Wake et al., 1989). To investigate the distribution of Kupffer cells around infected hepatocytes, we stained liver sections with the Kupffer cell marker CLEC4F (Scott et al., 2016; Krenkel and Tacke, 2017; Guillot and Tacke, 2019) and visualized liver stage parasites using DAPI (**Figures 4A–C**). We observed an increase in CLEC4F^+^ cells surrounding the parasite, with elevated density in Ring 1 compared to Ring 2, bystander cells within the same liver, and an identical-sized area of tissue within uninfected animals (**Figure 4D**). Interestingly, CLEC4F^+^ Kupffer cells often appear to wrap themselves around the LS-infected hepatocyte (**Figure 4B**). We then asked if high Kupffer cell density around infected cells at 44 hpi could be due to selection of an existing microenvironment at the time of hepatocyte invasion, or due to cells migrating to the site after infection had been established. Although often referred to as “resident”, Kupffer cells have been shown to migrate along sinusoids within the liver (mean of 4.6µm/min) (MacPhee et al., 1992). When we quantified Kupffer cells around *P. yoelii* parasites in livers collected 24 hpi, we observed no statistically significant difference between the number of cells in Ring1 and Ring2 (**Figure 4E**). Because parasites are much smaller at 24hpi than 44hpi, with average diameters of 10µm and 45µm respectively, we also measured Kupffer cell distance from the parasite membrane. The most notable difference in distribution between 24 and 44hpi was the increase in Kupffer cell density within 40µm of the parasite membrane (**Figure 4F**). To further explore the hypothesis that the parasite is surrounded by Kupffer cells that have migrated to Ring 1 between 24 and 44hpi, rather than a shifting of cells due to the increase in hepatocyte mass that occurs as a result of LS growth, we compared the average number of Kupffer cells within 5µm from the membrane of the 44h parasite and 22.5µm from the membrane of the 24h parasite (55µm diameter ROIs) (**Figure S3**). Despite the increased potential area that could be occupied by Kupffer cells within the 24h ROI due to the smaller volume occupied by the parasite, there were ~4.4x more Kupffer cells within the 44h ROI. This difference was maintained when the ROI diameter was expanded to 65µm (**Figure S3**), indicating that the increase in Kupffer cell density at 44hpi is not due to the expansion of the parasite towards pre-existing cells within close proximity.

Finally, to evaluate if a Kupffer cell dense region was selected as part of the sporozoite traversal process that occurs prior to hepatocyte entry, we utilized the spect2- parasite strain. Wild type parasites enter the liver through a hepatocyte, Kupffer cell, or liver endothelial cell, and then traverse through several hepatocytes using a transient vacuole before finally invading a final hepatocyte within a parasitophorous vacuole (Mota et al., 2001; Frevert et al., 2005; Tavares et al., 2013). Spect2^-^ parasites that do not successfully invade are phagocytosed by Kupffer cells or fail to egress from their transient vacuole and are eliminated by host cell lysosomes (Ishino et al., 2005; Risco-Castillo et al., 2015; Yang et al., 2017). This inability to traverse multiple hepatocytes limits their ability to travel through many cells; this may impact the parasite’s ability to select a particular local microenvironment. We infected mice with the spect2^-^ *P. yoelii* strain and measured Kupffer cell density at 44hpi. The pattern of Kupffer cell density around infected hepatocytes was maintained for spect2^-^ infected livers (**Figure 4G**), indicating that cell traversal does not influence Kupffer cell density around the infected cell.

We next sought to investigate the molecular characteristics of parasite-surrounding Kupffer cells. We revisited the DSP data (**Figure 3** and **Table S4**), and calculated pairwise Pearson’s correlation coefficients between area normalized signal in Ring1 for all antibodies that were upregulated in Ring1 when compared to Ring2 (Groups 1,4 and 5, **Figure 3C**). We reasoned that if levels of two or more of these proteins correlated strongly with each other they might be linked to a particular cell type or process that distinguishes the microenvironment that surrounds infected hepatocytes. Using Pearson’s correlation coefficients, we identified subsets of proteins that correlated with each other (**Figure S2**). The strongest correlations were between B7H3, CD163, and Src, all of which are expressed by Kupffer cells and have been linked to tolerogenic M2 polarization of macrophages, particularly within the tumor microenvironment (Sun et al., 2012; Kang et al., 2015; Mao et al., 2017; Ge and Ding, 2020).

**Figure 5:**
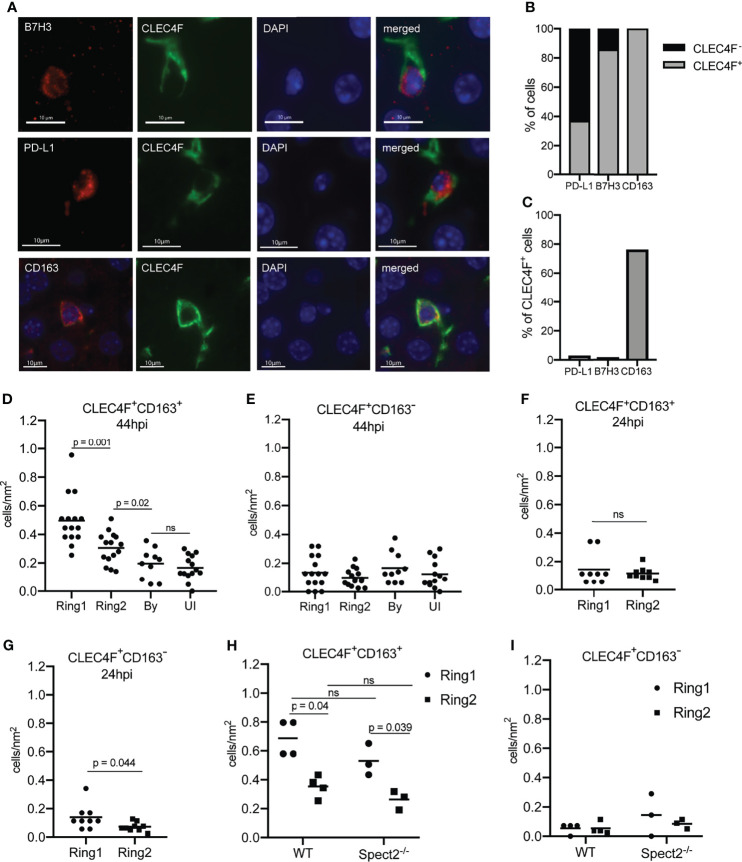
Tolerogenic macrophages migrate to surround *Plasmodium* infected hepatocytes. **(A)** Representative images of fluorescent staining of infected tissue for B7H3, PD-L1, and CD163. **(B)** Proportion of PD-L1^+^, B7H3^+^, and CD163^+^ cells within rings around infected cells that were CLEC4F^+^ or CLEC4F^-^. **(C)** Proportion of CLEC4F+ cells within rings around infected cells that were PD-L1^+^, B7H3^+^, or CD163^+^. **(D)** Number of CD163^+^CLEC4F^+^ and **(E)** CD163^-^CLEC4F^+^ cells normalized by area in Ring1, Ring2, bystander (By) tissue, and uninfected (UI) tissue at 44hpi. 3-6 matched ROI sets were counted from each of three mice. **(F)** Number of CD163^+^CLEC4F^+^ and **(G)** CD163^-^CLEC4F^+^ cells normalized by area at 24hpi. **(H)** Number of CD163^+^CLEC4F^+^ and **(I)** CD163^-^CLEC4F^+^ cells normalized by area, in Ring1 and Ring2 around wild type (WT) and Spect2^-/-^ parasites at 44hpi. ns, not significant.

We asked if the correlated proteins were expressed in overlapping populations of cells in Ring1 and Ring2. We found that CD163 was exclusively, and B7H3 almost exclusively, expressed on CLEC4F^+^ cells (**Figures 5A, B**). We also stained for PD-L1, which was part of both antibody panels and consistently upregulated in Ring1 compared to Ring2 but did not correlate with B7H3 and CD163. Of note, though they showed similar trends when averaged across multiple ROIs (**Table S4**), the two PD-L1 antibodies did not correlate well with each other (**Figure S2**). This could be due to differences between the two tissue sections that were used for each antibody panel or to different binding efficiencies of the two antibodies which are not identical. PD-L1 was found on both CLECF4^+^ and CLEC4F^-^ cells within Ring 1, with over 60% of PD-L1^+^ cells not expressing CLEC4F (**Figures 5A, B**). This is consistent with its lack of correlation with CD163 and B7H3, as well as published studies demonstrating PD-L1 expression on a variety of immune cell types (Wu et al., 2019). B7H3^+^ and PD-L1^+^ Kupffer cells were very rare, 1.6% and 2.9% of all CLEC4F^+^ cells, respectively, however CD163^+^ cells were abundant and represented a majority of CLEC4F^+^ cells (**Figure 5C**).

Quantification of CD163^+^ Kupffer cells revealed that more CD163^+^CLEC4F^+^, but not CD163^-^CLEC4F^+^, cells were present in Ring1 compared to Ring2, bystander, and uninfected tissue regions at 44hpi (**Figures 5D, E**). Interestingly, increased numbers of CD163^+^ Kupffer cells were not found around *P. yoelii* parasites in livers collected 24 hpi (**Figure 5F**), but CLEC4F^+^CD163^-^ cells were slightly elevated in Ring1 compared to Ring2 (**Figure 5G**). Finally, we compared CD163 expression in Ring1 and Ring2 around WT and spect2-- parasites. CLEC4F^+^CD163^+^ cells were present at a higher density in Ring1 compared to Ring2 in both WT and Spect2- infected livers, with no difference between parasite strains (**Figure 5H**). CLEC4F^+^CD163^-^ cell levels were not significantly different between rings or between parasite strains (**Figure 5I**).

## Discussion

In this study we utilized digital spatial profiling to characterize host total and phosphorylated proteins in and around *Plasmodium*-infected hepatocytes *in vivo.* Doing these analyses while preserving the tissue architecture allowed us to link a specific microenvironment to infected cells. Probing a large panel of proteins simultaneously in the same tissue regions allowed us to investigate the molecular characteristics of parasite-surrounding cells. Importantly, this is challenging to do with conventional approaches as it requires candidate-based investigation into specific candidate markers that may or may not be relevant for the cell type of interest. By measuring changes in host proteins in concentric rings around infected hepatocytes and correlations between these proteins we identified an influx of CD163^+^ Kupffer cells towards the parasite during the second half of liver stage infection.

Kupffer cells originate from fetal liver erythromyeloid progenitors and in the adult liver, under resting conditions, their populations are self-renewing independent of bone marrow-derived cells (Gomez Perdiguero et al., 2015). Upon Kupffer cell depletion, infiltrating circulating monocytes differentiate into Kupffer cells starting 96 hours post-depletion (Scott et al., 2016). Notably, monocyte-derived Kupffer cells do not begin expressing CLEC4F until between 72-96 hours post-depletion (Scott et al., 2016), indicating that the increase in CLEC4F^+^ cells we observed near the parasite between 24 and 44hpi is highly unlikely to be due to differentiation of infiltrating monocytes. More likely, resident CLEC4F^+^ cells that have migrated towards the parasite.

Macrophages exist along a continuum of states that are often described as ranging from pro-inflammatory (M1) to tolerogenic (M2) (Guillot and Tacke, 2019). Alterations of Kupffer cells upon sporozoite exposure are consistent with a model that parasites manipulate Kupffer cells to produce a tolerogenic environment for their development within the liver. Kupffer cell exposure to sporozoites has been shown to suppress respiratory burst (Usynin et al., 2007), suppress antigen presentation (Steers et al., 2005), and skew cytokine production upon pro-inflammatory stimulation towards an anti-inflammatory response (Klotz and Frevert, 2008). Co-culture of CD8^+^ T cells with sporozoite-stimulated monocyte-derived macrophages also produced less IFN*ν* (Winkel et al., 2020). Most of these studies were conducted with prolonged co-incubation of sporozoites and macrophages *in vitro.* Several functional studies have been conducted in which Kupffer cells are depleted before sporozoite infection (Vreden et al., 1993; Baer et al., 2007), but the importance of these cells for infection maintenance are confounded by the effects of depletion on hepatocyte invasion.

CD163 is commonly utilized as a tolerogenic macrophage marker (Guillot and Tacke, 2019), however it may also play a functional role in maintenance of LS infection. CD163 is a scavenger receptor expressed on monocytes and macrophages that binds and facilitates the internalization and clearance of hemoglobin-haptoglobin (HbHp) complexes, thereby protecting the liver from oxidative damage (Van Gorp et al., 2010). Binding of HbHp complexes promotes the expression of heme oxygenase-1 (HO-1) which degrades the Hb heme subunit, producing biliverdin, iron, and carbon dioxide. Although not part of the panel we interrogated in this study, HO-1 has been shown to be upregulated in macrophages and hepatocytes during *Plasmodium* LS infection and to be critical for infection maintenance (Epiphanio et al., 2008). Of particular interest, HO-1 was not found to be essential for *Plasmodium* LS infection when hepatocytes are cultured alone *ex vivo*, suggesting its effect on nonparenchymal cells influences infection. Higher expression of CD163 on Kupffer cells has also been linked to greater phagocytic activity (He et al., 2009). Merozoite forms (often as ‘packages’ of parasites called merozomes) (Sturm et al., 2006) of the parasite exit the infected hepatocyte and enter the blood stream between 50-52 hpi. It is intriguing to speculate that the wrapping of Kupffer cells around infected hepatocytes (**Figure 4C**) late during infection could facilitate phagocytosis of the hepatocyte by Kupffer cells after merozoite exit. By regulating antigen presentation and inflammation around the infected cell microenvironment, Kupffer cells could be influencing the development of subsequent immunity. This could have long-reaching consequences not only for infection, but also for the development of whole parasite vaccines.

We are unable to determine from our data if CD163^+^ Kupffer cells are infiltrating in towards the parasite, or if they begin expressing CD163 upon gaining their location near the infected cell, however the small increase in CD163^-^CLEC4F^+^ cells in Ring1 compared to Ring2 at 24 hpi (**Figure 5G**) supports the latter hypothesis. PD-L1 expression, which is increased in Ring1 compared to Ring2, has been shown to be induced in monocyte-derived macrophages in the skin upon exposure to *Plasmodium* sporozoites (Winkel et al., 2020). A portion of sporozoites are thought to cross and interact with Kupffer cells as they are entering the liver (Tavares et al., 2013), however, as no difference in CD163^+^ Kupffer cell density was observed between WT parasites and Spect2^-^ parasites, which are traversal deficient, we hypothesize that the increase in CD163 expression is not present prior to sporozoite invasion of hepatocytes.

By utilizing DSP we were able to measure host protein on a large scale in single infected hepatocytes *in vivo* without sorting cells and without dissociating cells from their microenvironment. While we cannot rule out non-specific binding of antibodies to parasite proteins, the minimal sample processing may better preserve the host cell condition compared to experiments that require hours of cell sorting, particularly in the case of post-translational modifications. Several of the proteins up-regulated in infected cells were phosphorylated, indicating increased activity: p-IK-Ba, p-S6, and p-Erk. Consistent with our results, Erk (MAPK1) activity was previously identified by our lab as important for maintenance of *P. yoelii* infection *in vitro* using a kinase inhibitor screen combined with a machine learning algorithm (Arang et al., 2017). Additionally, levels of p-S6 are higher in hepatocytes that are more susceptible to *Plasmodium* infection and in infected hepatocytes *in vitro*, although in the context of infection S6 phosphorylation is dysregulated from classical upstream activator p-Akt (Glennon et al., 2019). One surprising result was the increase seen in p53 levels in infected cells. *In vitro*, early during infection, P53 is suppressed in infected hepatocytes. Boosting levels of P53 efficiently eliminates liver stages parasites both *in vitro* and *in vivo.* The difference in time point (44h in this study, as opposed to 24h in previous *in vitro* studies) could explain the observed difference in p53 levels (Kaushansky et al., 2013a). It will be interesting to explore if the increase in p53 seen here at 44hpi represents a loss of regulation by the parasite as it shifts towards merozoite production and preparation for egress.

A very small number of parasites successfully invade hepatocytes and complete LS infection. This, and the extensive remodeling of infected hepatocytes, suggest *Plasmodium* parasites have substantial requirements of their host cells. By identifying proteins/post-translational modifications that show very little variation among infected compared to uninfected cells, we may be able to identify specific targets or signaling nodes that are maintained within, or selected for, very narrow limits by the parasite. These factors could represent promising drug targets, as even small perturbations of these factors could have dire consequences for the developing LS parasite. Future studies might also explore the signaling perturbations that are altered in the context of liver stage parasites that do not complete development, as is the case with irradiated, drug-killed or genetically attenuated parasites. While this study is entirely focused on the rodent parasite *Plasmodium yoelii*, DSP is readily adaptable to the study of human-infectious species *Plasmodium falciparum* and *Plasmodium vivax* using a humanized mouse model (Vaughan et al., 2012; Mikolajczak et al., 2015) or other tissue models. Evaluating the spatially resolved host transcriptomic and proteomic responses that occur after infection, particularly in the context of the dormant *P. vivax* hypnozoite, may reveal novel biology regulating infection maintenance and development of immunity.

## Methods

### Mosquito Rearing and Sporozoite Production

Female 6–8-week-old Swiss Webster mice (Envigo) were injected with blood stage *Plasmodium yoelii* 17XNL parasites. Infected mice were used to feed female *Anopheles stephensi* mosquitoes after gametocyte exflagellation was observed. Salivary gland sporozoites were isolated according to the standard procedures at days 14 or 15 post blood meal. Animal handling was conducted according to the Institutional Animal Care and Use Committee-approved protocols.

### Mouse Infections

6-8-week-old female Balb/cAnN mice were purchased from Envigo. All mice were maintained in accordance with protocols approved by Seattle Children’s Research Institute Institutional Animal Care and Use Committee (IACUC00502). Mice were infected by retro orbital injection with 100,000 or 1 million *P. yoelii* sporozoites. Livers from infected, or uninfected age-matched, mice were harvested at 24 or 44 hpi and fixed in 4% paraformaldehyde for 24 hours. Tissues were then paraffin embedded, cut into 4mm sections, and mounted on positively charged glass slides. Mounted liver slices were then used for digital spatial profiling or immunofluorescence staining.

### Digital Spatial Profiling

Digital spatial profiling (DSP) was performed by NanoString Technologies using the GeoMx Digital Spatial profiler. For selecting regions of interest, slides were stained with DAPI and a fluorescent conjugated antibody against *Py*HSP70. Slides were simultaneously incubated with one of two pre-validated panels of 42-43 oligo-tagged antibodies (**Table S1**). Counts were normalized to an internal control (ERCC) and to ROI area. Data were not normalized to nucleus counts as *Plasmodium* parasites have been shown to preferentially invade highly polyploid hepatocytes (Austin et al., 2014). Traditional “housekeeping” proteins (RPS6, alpha-tubulin, histoneH3) within the antibody panels were also not used for normalization as they have either been previously identified to increase in infected cells, show cross-reactivity, or were upregulated in infected cells in our data (Mikolajczak et al., 2015; Glennon et al., 2019). Data normalized only to internal controls (ERCC) are available in **Tables S1**, **S3**. Data were analyzed by ANOVA with paired or unpaired multiple comparisons as appropriate. Our single infected-cell ROIs may encompass a portion of neighboring cells.

### Immunofluorescence Staining

Slide-mounted liver slices were washed twice in xylene for 3 minutes followed by washes in 100%, 95%, 70% and 50% ethanol for 3 minutes each. Slides were then washed with DI water and heated to 90C for 30 minutes in 1% citrate-based antigen unmasking solution (Vector Laboratories) using a Biocare Medical Decloaking Chamber. Slides were washed with TBS-0.025% Tween (TBST) and then blocked for 4 hours in TBST containing 1.5% BSA and 15% goat serum (Sigma Aldrich). Slides were incubated in primary antibodies at 4C overnight. Following primary antibody staining, slides were washed with TBST and incubated with secondary antibodies and DAPI (1:3,000) for 1 hour at room temperature. Slides were washed with TBST and autofluorescence quenched using Vector TrueView (Vector Labs). Fluoromount G mounting media was used to preserve fluorescence signal. Primary antibodies were used at the following concentrations: *Py*Hsp70 1:1,000, *Py*CSP-488 1:500, p-p44/42 1:200 (Cell Signaling 4370), p-IK-Ba 1:200 (Cell Signaling 2850), p-Akt 1:100 (Cell Signaling 9271), CD163 1:500 (Proteintech 16646-1-AP), CLEC4F-647 1:100 (BioLegend 156804), PD-L1 1:200 (Cell Signaling 64988), B7H3 1:200 (Novus Bio NB600-1441). Secondary antibodies anti-mouse AlexaFluor-488, anti-rabbit AlexaFluor-594, and anti-rabbit AlexaFluor-647 (Invitrogen) were used at a 1:1,000 dilution.

### Imaging and Quantification

Images (40X) were acquired using a DeltaVision Elite High Resolution Microscope. Z-stacks of 0.3*μ*m thickness were taken for images encompassing infected and uninfected cells. For cell quantification within Ring ROIs 3x3 image panels were taken with a 60-pixel overlap. Images were stitched and deconvolved using the DeltaVision Softworx software and were visualized using Imaris software. ImageJ was used to quantify fluorescence intensity within defined ROIs. Distances from parasites to Kupffer cells were measured between nucleus centers, or from the parasite membrane to the Kupffer cell nucleus, using Imaris software. Only Kupffer cells with a visible, stained nucleus (DAPI) were included in counts.

The phosphosignaling network was reconstructed using PhosphoSitePlus^®^, a curated knowledgebase dedicated to mammalian post-translational modifications (https://www.phosphosite.org) (Hornbeck et al., 2015).

## Data Availability Statement

The original contributions presented in the study are included in the article/**Supplementary Material**. Further inquiries can be directed to the corresponding author.

## Ethics Statement

The animal study was reviewed and approved by Seattle Children’s Research Institute Institutional Animal Care and Use Committee.

## Author Contributions

EG and AK designed experiments. EG, TT, and VP conducted experiments. Data analysis was done by EG, LW, TT, and SR. All authors contributed to writing and editing the manuscript.

## Funding

This work was funded by grants R01GM101183, R21AI151344, and R01AI158719 from the National Institutes of Health to AK. SR is the recipient of T32 training grant 5T32HD007233-39 from the University of Washington and the National Institutes of Health.

## Conflict of Interest

The authors declare that the research was conducted in the absence of any commercial or financial relationships that could be construed as a potential conflict of interest.

## Publisher’s Note

All claims expressed in this article are solely those of the authors and do not necessarily represent those of their affiliated organizations, or those of the publisher, the editors and the reviewers. Any product that may be evaluated in this article, or claim that may be made by its manufacturer, is not guaranteed or endorsed by the publisher.

## Supplementary Material

Supplementary Figure 1 DSP results are reproducible across runs and between pooled and single ROIs. Average fold change between infected and uninfected ROIs for each antibody from **(A)** two independent DSP runs, and **(B)** two slides run at the same time. Data were analyzed by linear regression. **(C)** For each antibody the average area-normalized signal 9 single infected ROIs was plotted against that of one pooled infected ROI, all from the same mouse. **(D)** For each antibody the average area-normalized signal 9 single uninfected ROIs was plotted against that of one pooled uninfected ROI. Data were analyzed by linear regression. **(E)** Representative images of uninfected regions total magnification of 400x at 44hpi.

Supplementary Figure 2 A subset of upregulated (phospho)proteins in proximity to *Plasmodium*-infected hepatocytes are correlated. **(A)** Heat map indicating the Pearson correlation coefficient for each pair of antibodies for those significantly upregulated in Ring1 compared to Ring2. Antibodies from multiple panels are identified as part of panel **(A)** or **(B)**. n = 6 matched ROI sets from a single mouse.

Supplementary Figure 3 Growth of *Plasmodium* infected hepatocyte does not account for increased Kupffer cell density around cell. Kupffer cell density within circular ROIs of 55µm and 65µm around parasites at 24hpi and 44hpi. Parasites are shown as green circles. Length of lines is indicated in microns. Circles and rings are shown to scale. Kupffer cell density is shown as the mean from 3-4 parasites per mouse from 3 mice per time point.
