# Supplementary figures and images for: Elucidating Spatially-Resolved Changes in Host Signaling During Plasmodium Liver-Stage Infection
Source: Front Cell Infect Microbiol. 2022 Jan 17;11:804186. doi: 10.3389/fcimb.2021.804186 (PMC8801743; doi:10.3389/fcimb.2021.804186)

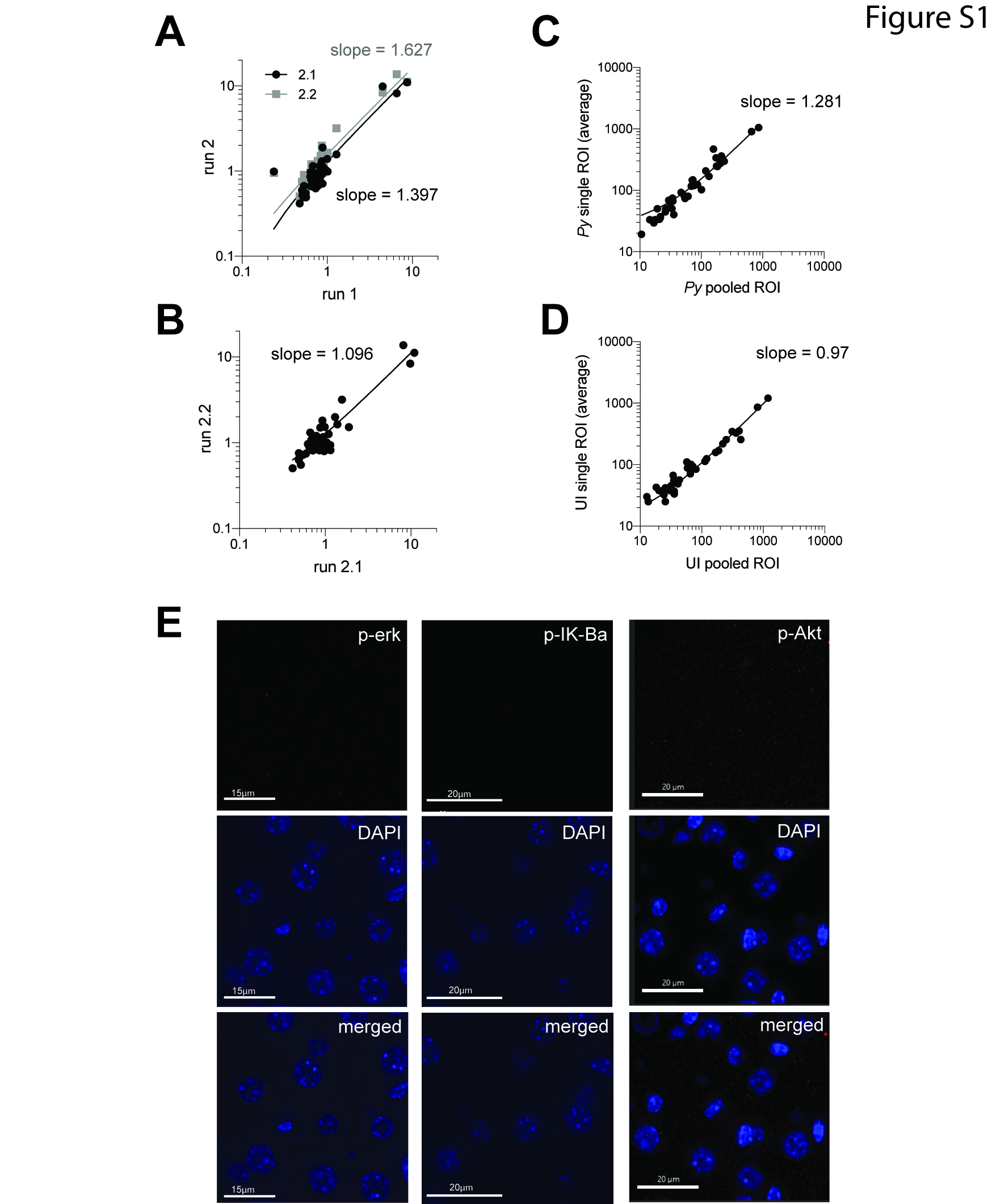

Supplement: Supplementary Figure 1 — DSP results are reproducible across runs and between pooled and single ROIs. Average fold change between infected and uninfected ROIs for each antibody from (A) two independent DSP runs, and (B) two slides run at the same time. Data were analyzed by linear regression. (C) For each antibody the average area-normalized signal 9 single infected ROIs was plotted against that of one pooled infected ROI, all from the same mouse. (D) For each antibody the average area-normalized signal 9 single uninfected ROIs was plotted against that of one pooled uninfected ROI. Data were analyzed by linear regression. (E) Representative images of uninfected regions total magnification of 400x at 44hpi. [file Image_1.tif]

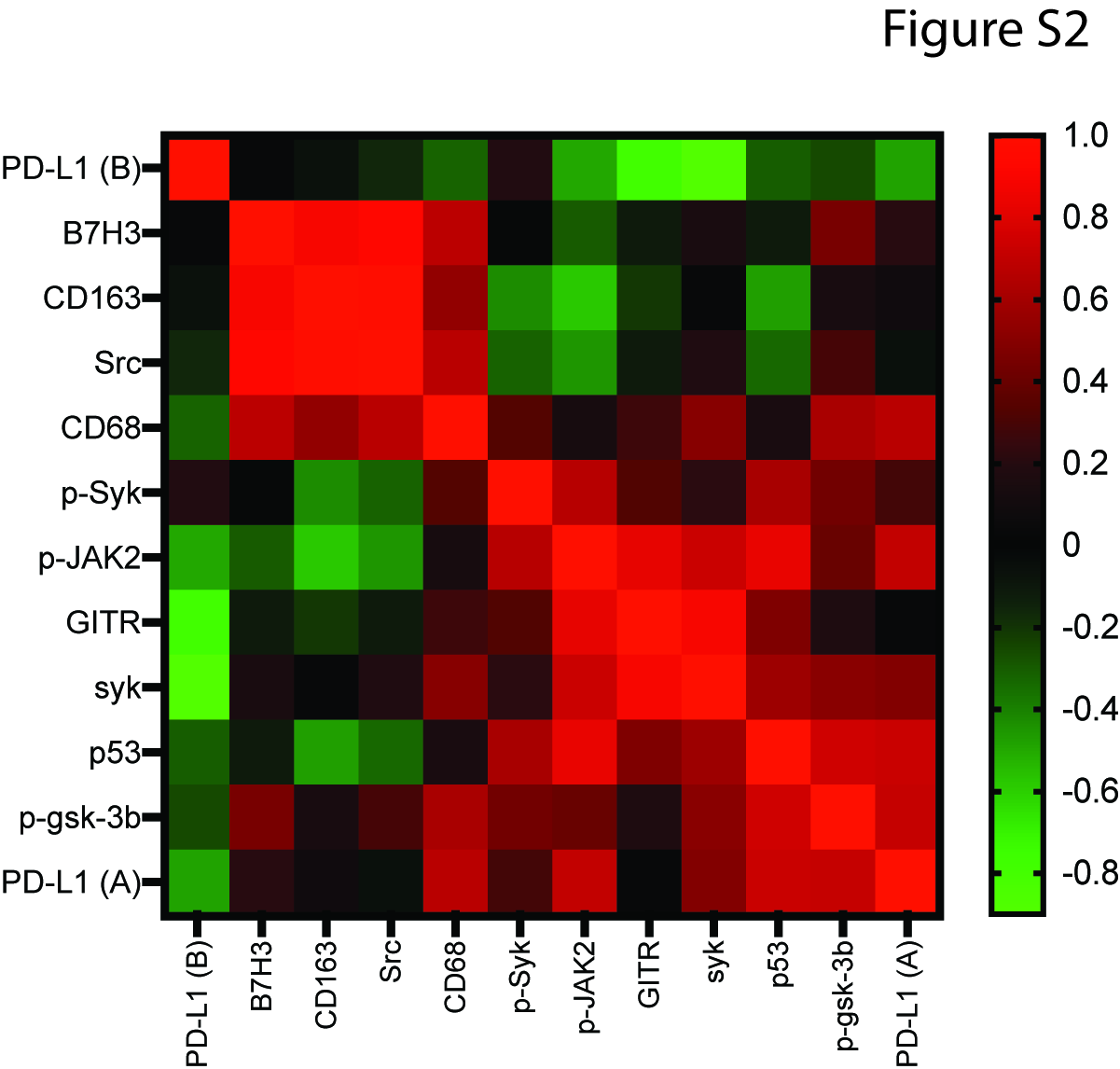

Supplement: Supplementary Figure 2 — A subset of upregulated (phospho)proteins in proximity to Plasmodium-infected hepatocytes are correlated. (A) Heat map indicating the Pearson correlation coefficient for each pair of antibodies for those significantly upregulated in Ring1 compared to Ring2. Antibodies from multiple panels are identified as part of panel (A) or (B). n = 6 matched ROI sets from a single mouse. [file Image_2.tif]

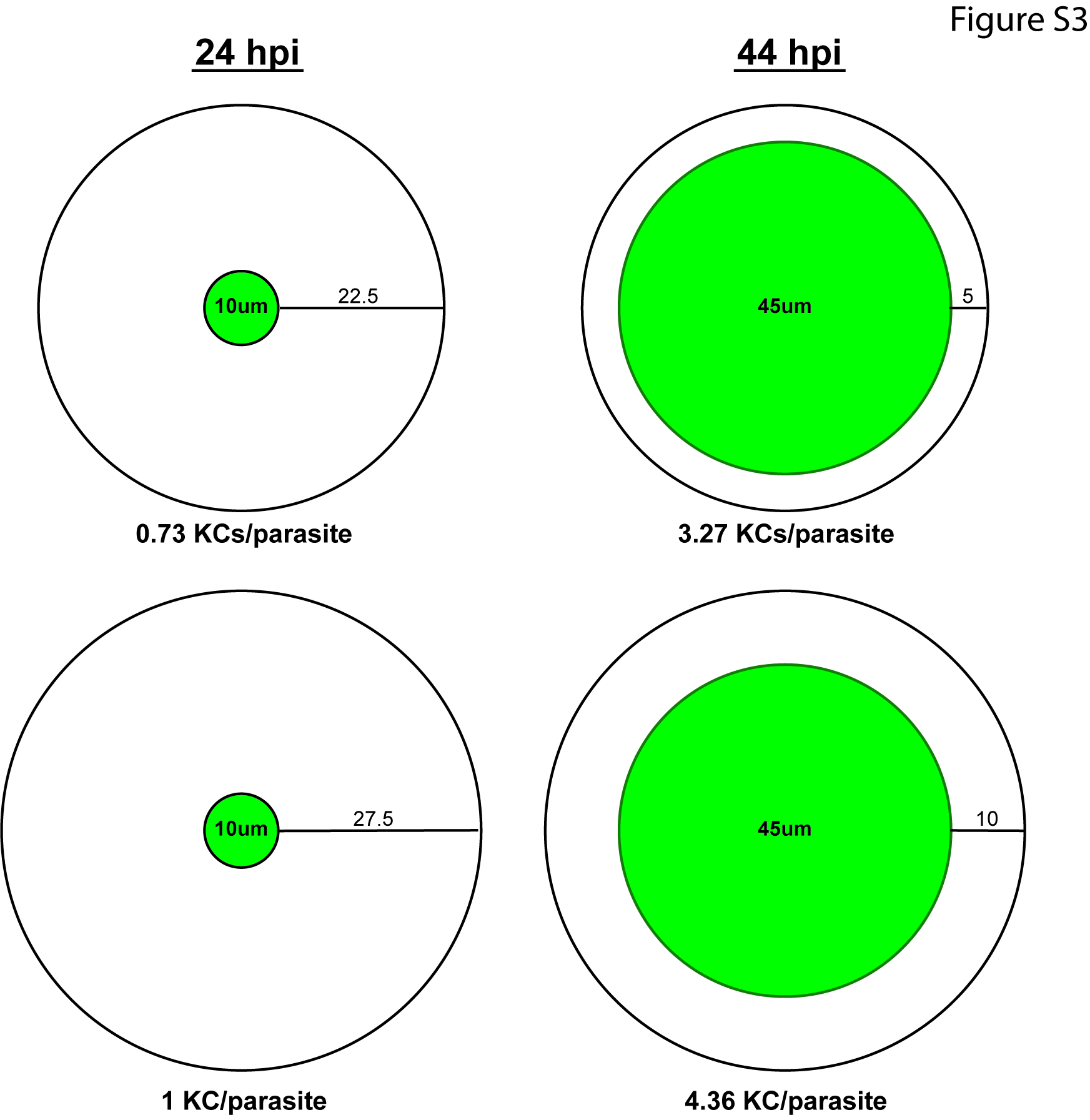

Supplement: Supplementary Figure 3 — Growth of Plasmodium infected hepatocyte does not account for increased Kupffer cell density around cell. Kupffer cell density within circular ROIs of 55µm and 65µm around parasites at 24hpi and 44hpi. Parasites are shown as green circles. Length of lines is indicated in microns. Circles and rings are shown to scale. Kupffer cell density is shown as the mean from 3-4 parasites per mouse from 3 mice per time point. [file Image_3.tif]
